# Salinity stress endurance of the plants with the aid of bacterial genes

**DOI:** 10.3389/fgene.2023.1049608

**Published:** 2023-04-17

**Authors:** Seyyedeh Maryam Zamanzadeh-Nasrabadi, Fatemeh Mohammadiapanah, Mehdi Hosseini-Mazinani, Sajjad Sarikhan

**Affiliations:** ^1^Pharmaceutial Biotechnology Lab, School of Biology and Center of Excellence in Phylogeny of Living Organisms, College of Science, University of Tehran, Tehran, Iran; ^2^ National Institute of Genetic Engineering and Biotechnology, Tehran, Iran; ^3^ Molecular Bank, Iranian Biological Resource Center (IBRC), ACECR, Tehran, Iran

**Keywords:** salinity stress alleviation, PGPB (plant growth-promoting bacteria), gene functions and pathways, molecular mechainsm, salinity stress, drought stress

## Abstract

The application of plant growth-promoting bacteria (PGPB) is vital for sustainable agriculture with continuous world population growth and an increase in soil salinity. Salinity is one of the severe abiotic stresses which lessens the productivity of agricultural lands. Plant growth-promoting bacteria are key players in solving this problem and can mitigate salinity stress. The highest of reported halotolerant Plant growth-promoting bacteria belonged to *Firmicutes* (approximately 50%), *Proteobacteria* (40%), and *Actinobacteria* (10%), respectively. The most dominant genera of halotolerant plant growth-promoting bacteria are *Bacillus* and *Pseudomonas*. Currently, the identification of new plant growth-promoting bacteria with special beneficial properties is increasingly needed. Moreover, for the effective use of plant growth-promoting bacteria in agriculture, the unknown molecular aspects of their function and interaction with plants must be defined. Omics and meta-omics studies can unreveal these unknown genes and pathways. However, more accurate omics studies need a detailed understanding of so far known molecular mechanisms of plant stress protection by plant growth-promoting bacteria. In this review, the molecular basis of salinity stress mitigation by plant growth-promoting bacteria is presented, the identified genes in the genomes of 20 halotolerant plant growth-promoting bacteria are assessed, and the prevalence of their involved genes is highlighted. The genes related to the synthesis of indole acetic acid (IAA) (70%), siderophores (60%), osmoprotectants (80%), chaperons (40%), 1-aminocyclopropane-1-carboxylate (ACC) deaminase (50%), and antioxidants (50%), phosphate solubilization (60%), and ion homeostasis (80%) were the most common detected genes in the genomes of evaluated halotolerant plant growth-promoting and salinity stress-alleviating bacteria. The most prevalent genes can be applied as candidates for designing molecular markers for screening of new halotolerant plant growth-promoting bacteria.

## 1 Introduction

Currently, climate change is the principal menace to sustainable agriculture. Biotic (30%) and abiotic (50%) stresses are the main restrictions for agriculture (Kumar and Verma, 2018; Sharma A. et al., 2021; Poria et al., 2022). Over 20% of the cultivable soil worldwide is influenced by salinity stress, and every year, about 1%–2% of arable lands are disqualified by the increased salinity (Arora et al., 2020; Noori et al., 2021). Salinity and drought as the most destructive abiotic stresses are causing secondary detrimental effects, including oxidative and osmotic stresses shared with both stresses; besides ionic stress in salinity (Chaudhry et al., 2022). Salinity affects plants at morphological, physiological, biochemical, and molecular levels. Salinity causes the decrease of the leaf area, and chlorophyll content of leaves, leaf thickening, decreased shoot and root weight, necrosis of plants, wilting, drying, the reduction in seed germination, seedling growth, flowering, and fruiting, less grain weight, oxidative damage, electrolyte leakage, reduced carbon fixation, membrane damage, loss of organelle function, closure of stomata, nutrient imbalance, reduction of photosynthesis, and phytohormones production (Saberi Riseh et al., 2021; Ullah et al., 2021). Different strategies are employed to ameliorate the crop resistance to stress, comprising breeding, genetic engineering, CRISPR/Cas9 technology, chemical priming, and biological priming (Godoy et al., 2021; Wang et al., 2023). The application of PGPB is cost-effective and the most capable strategy following the challenges related to the development of new tolerant plants due to the intricacy of abiotic stress tolerance mechanisms, the awareness of the toxicity of agrochemicals, and alternating green technologies (Mohammadipanah and Zamanzadeh, 2019; Alberton et al., 2020; Jiang et al., 2021; Saeed et al., 2021). Moreover, although beneficial effect of biochar amendments in agriculture have been demonstrated, biochar addition may not certainly play a positive role for all soils type, climate, and plants species, high rates application of biochar may have side effects on weed control, delay in flowering (Alberton et al., 2020), and biochar adsorbs essential nutrients such as nitrogen and Fe (Kavitha et al., 2018; Wang et al., 2020).

**FIGURE 1:**
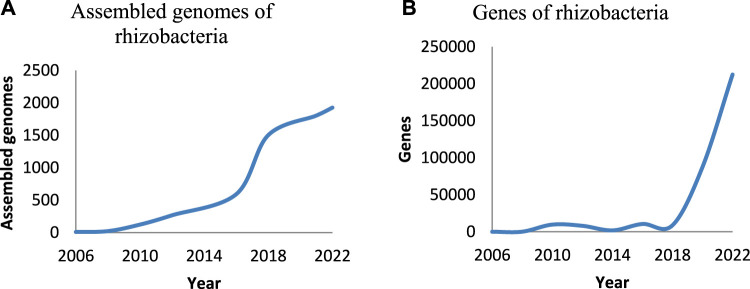
The studies on genomes and genes of rhizobacteria per year. The number of the genomes of rhizobacteria that were assembled **(A)**, the number of represented genes of rhizobacteria **(B)** (information was.

Among the assessed halotolerant PGPB in 40 articles, 54% belonged to *Firmicutes*, 39% to *Proteobacteria*, and 7% to *Actinobacteria*. The most dominant genera of halotolerant PGPB are *Bacillus* and *Pseudomonas*. PGPB are capable of offering cross-protection against several stresses and increasing plant growth *via* different direct and indirect mechanisms, including modification of root morphology, nutrient attainment, synthesis of exopolysaccharides, phytohormones, volatile compounds, and 1-aminocyclopropane-1-carboxylate (ACC) deaminase, ion homeostasis, inducing aggregation of antioxidants and compatible solutes, induced systemic tolerance, and modulation of the stress-responsive genes (Mohammadipanah and Zamanzadeh, 2019; Leontidou et al., 2020). Despite extensive investigation on the mechanisms of action of PGPB, information on the molecular aspect of these mechanisms is slight (Balasubramanian et al., 2021). Applying next-generation sequencing (NGS), computational tools, and omics methods (meta (genomics) (transcriptomics) (proteomics) and metabolomics) can integrate data on molecular aspects of the plant-microbe reciprocal action or effect (Meena et al., 2017; Shelake et al., 2019). In recent years, the studies on molecular features of rhizobacteria have been increasing (Figure 1). However, there are many bacteria whose plant growth-promoting capabilities have been proven in the laboratory, pot, and field, but their genomes and molecular aspects of action have not been investigated. By assaying the genomes of these bacteria, new pathways and genes may be identified. Knowing the genes and pathways related to stress mitigation, which are identified so far, are essential for omics and meta-omics studies. In addition, the assignment of related gene sets to PGPB action assists in designing biomarkers of rapid screening of efficient plant growth-promoting strains. There are many whole genomes of the environmental bacteria, which can be screened by applying molecular markers to identify new plant growth-promoting taxa.

While many studies have presented the mechanisms of action of PGPB, there is not any systematic review on genes conferring the plant growth-promoting trait to halotolerant symbiotic bacteria. In this survey, the molecular aspects of the action mechanisms of halotolerant PGPB are expressed. In addition, the identified pathways and genes related to plant growth promotion and salinity stress alleviation in the genomes of 20 halotolerant PGPB, including *Pseudomonas fluorescens* PCL1751 (Cho et al., 2015), *Cronobacter muytjensii* JZ38 (Eida et al., 2020), *Klebsiella* sp. KBG6.2 (Girma et al., 2020), *Enterobacter roggenkampii* ED5 (Guo et al., 2020), *Jejubacter calystegiae* (Jiang et al., 2021), *Klebsiella* sp. D5A (Liu et al., 2016), *Bacillus megaterium* STB1 (Nascimento et al., 2020), *Pseudomonas thivervalensis* SC5 (Nascimento et al., 2021), *Pantoea agglomerans* ANP8 (Noori et al., 2021), *Hartmannibacter diazotrophicus* E19T (Suarez et al., 2019), *Bacillus flexus* KLBMP 4941 (Wang et al., 2017), *Enterobacter* sp. SA187 (Andres-Barrao et al., 2017), *Bacillus paralicheniformis* ES-1 (Iqbal et al., 2022), *Brevibacterium sediminis* MG-1 (Lutfullin et al., 2022), *Bacillus* sp. BH32 (Belaouni et al., 2022), *Pseudomonas chloritidismutans* 6L11 (Zhou et al., 2022), *Stenotrophomonas rhizophila* IS26 (Dif et al., 2022), *Pseudomonas* sp. UW4 (Duan et al., 2013), *Achromobacter xylosoxidans* SQU-1 (Jana and Yaish, 2021), and *Stenotrophomonas* 169 (Ulrich et al., 2021) are assessed and these genes prevalence are characterized. Moreover, the studies conducted on the assay of transcriptome, proteome, and metabolome of PGPB in interaction with plants are presented.

For this review, an extensive search from the National Center for Biotechnology Information (bookshelf, PubMed, Assembly, BioProject, BioSample, and genome), Google Scholar, Scopus, ScienceDirect, and Springer was performed using relevant keywords such as halotolerant PGPB, salinity stress alleviation, PGPB genome mining, genome analysis, PGPR molecular action, PGPR pathways and genes, halotolerant rhizobacteria, genomes of rhizobacteria, genes of rhizobacteria, genome analysis tools, PGPB transcriptome, PGPB proteome, PGPB metabolome, and plant-bacteria interaction.

## 2 Omics methods for assaying molecular mechanisms of PGPB

Omics and meta-omics methods (genomics, transcriptomics, proteomics and metabolomics) help decipher molecular mechanisms of PGPB action to reveal the intricate plant–bacteria interactions. It is essential to understand how bacterial metabolites affect plant–bacteria interactions. Genomic analysis of 20 halotolerant PGPB in this study present the related genes to plant-bacteria interaction in the genomes of these bacteria.

Metagenomic studies of bacterial communities provide the basic evidence of plants-bacteria interaction whichcan bepractical to be used in identifying novel species with special traits (Fadiji et al., 2022; Mukherjee, 2022). Multiple metagenomic analyses have been carried out for investigating the genes related to plant growth-promoting traits of bacterial community from soil samples of oil field at Wietze (Eze et al., 2021), from maize rhizosphere in the South Africa (Molefe et al., 2021), associated with the root of sugar beet (Tsurumaru et al., 2015), and the endophytes of *Emilia sonchifolia* (Linn.) DC (Urumbil and Anilkumar 2021). These metagenomic analyses showed structural and functional diversity of plant microbiomes and allowed identification of genes and taxa putatively related to plant growth promotion. Only one report is available on the metagenome of plant rhizospheric bacteria exposed to salinity stress. The metagenomic analysis of the rhizospheric microbial community of grapevine under salinity stress showed that the salinity stress tolerance of grapevines was associated to the composition and functions of the rhizospheric microbial community (Wang et al., 2023).

Studies on gene expression have mainly focused on the expression assay of genes in plants in interaction with PGPB (Sheibani-Tezerji et al., 2015). The expression of genes, proteome, and metabolome in different hosts while interacting with PGPB have been assessed in several studies (Wang et al., 2005; Kwon et al., 2016; Kazemi-Oskuei et al., 2018; Gamez et al., 2019; Yoo et al., 2019; Malviya et al., 2020; Gozalia et al., 2021; Kataoka et al., 2021; Li et al., 2021; Mellidou et al., 2021; Gao et al., 2022; Samaras et al., 2022; Wiggins et al., 2022; Xu et al., 2022; Yadav et al., 2022).

However, only a limited number of studies described below have investigated the gene expression and metabolites in PGPB during interaction with plants. Transcriptome analysis of *Burkholderia phytofirmans* PsJN colonizing potato under drought stress showed the upregulation of genes related to transcriptional regulation, homeostasis, and the detoxification of ROS (Sheibani-Tezerji et al., 2015).The transcriptome analysis of plant growth-promoting *Paenibacillus polymyxa* YC0136 showed 286 genes were up-regulated and 223 genes were down-regulated under interaction with tobacco (Liu et al., 2020). The expression of genes was assayed in three new plant growth-promoting bacterial strains (two *Paenibacillus* sp. strains and one *Erwinia gerundensis* strain) in interaction with barley (Li et al., 2021). The transcriptome of the plant growth-promoting bacterium *Delftia acidovorans* RAY209 was assayed during interaction with soybean and canola roots (Suchan et al., 2020). The gene expression of *Azospirillum lipoferum* 4B during interaction with rice roots was assayed (Drogue et al., 2014). A metatranscriptomic study was conducted to investigate the contributions of different nitrogen-fixing bacteria present in the maize inoculated liquid (Gomez-Godinez et al., 2018). Metabolomics was applied to explore the exo-metabolome of three PGPB (*Pseudomonas putida* IDE-01, *Azospirillum brasilense* IDE-06, and *Bacilus megaterium* IDE-14) in interaction with maize and rice (Garcia et al., 2022). No twodimentional study was found on gene expression and metabolome of salinity stress-alleviating PGPB in interaction with plants. Metaphenome is the product of expressed functions encoded in microbial genomes and the environmentwhich consistes of themeta-omics technologies, including metagenomics, metatranscriptomics, metaproteomics, and metabolomics. The rhizosphere metaphenomics remains a significant challenge that needs to be addressed (Jansson and Hofmockel, 2018; Azeem et al., 2022).

Exploring the complexity of plant-soil-bacteria interactions allows the application of bacteria in an efficient manner to increase productivity of crops. Interactomics is a comprehensive technology for determining the pathways associated with communication between host plant and PGPB under environmental stresses which need simultaneous analysis the interactions between different biomolecules including proteins, and enzymes from both plants and bacterial cells. (Arora et al., 2020).

## 3 Molecular basis of the stress sensing and halo tolerance mechanisms of bacteria

Understanding molecular mechanisms related to stress tolerance in bacteria is vital for their application as plant stress protectors (Ahmed et al., 2018). Bacteria apply the cell surface extracytoplasmic function (ECF) sigma factors for sensing and responding to the surroundings. Mentioned signal transduction involves the outer membrane receptor, inner membrane-attached sigma factor regulator or anti-sigma factor, and ECF sigma factor. Anti-sigma factor firmly attaches to the ECF and maintains it inactive during lack of signal. In the presence of stress, the anti-sigma factor is decomposed. Consequently, the sigma factor is liberated and activates the expression of its related genes. Moreover, ECF sigma factors can be a factor in establishing the interactions between plants and bacteria (Sheibani-Tezerji et al., 2015).

The ability of osmotic stress mitigation in bacteria is evolved through horizontal gene transfer. The main adaptation mechanisms for tolerating salinity stress include ion homeostasis, accumulation of osmolytes, and production of universal proteins related to salt stress tolerance (Goyal et al., 2019). Na^+^/H^+^ antiporters are a group of transmembrane proteins that exist in the plasma membrane of nearly all cells and participate significantly in the conservation of intracellular pH, cellular sodium amount, homeostasis, and volume of the cell (Goyal et al., 2019). Five types of Na^+^/H^+^ antiporters are present in prokaryotes, including NhaA, NhaB, NhaC, NhaD, and NapA. Nhas perceive the ion concentration of the surroundings and moderate their action to preserve the homeostasis of cells (Kapoor and Kanwar, 2019). NhaA antiporter acts very selectively to exclude Na^+^ (Goyal et al., 2019). NhaA can be applied as an applicable marker to screen the salt tolerant strains (Kapoor and Kanwar, 2019). The genes of Nha proteins have been identified in the genomes of more than 80% of assessed halotolerant PGPB (Table 1).

**TABLE 1:** Identified genes involved in salt tolerance of 20 assessed halotolerant PGPB.

Product/Role	Genes	Bacteria	Assessed salinity concentration	References
**Heat-shock proteins (HSPs) synthesis**	*dnaJK, groEL, groES, htpGX*	*Klebsiella* sp. D5A	12% NaCl (w/v)	Liu et al. (2016)
	*htpGX, groL, groS, grpE, dnaK, and dnaJ*	*Bacillus paralicheniformis* ES-1	1.7 M NaCl	Iqbal et al. (2022)
	*dnaJK, groES, groEL, htpGX, hspQ, grpE, ibpA and clpB*	*Hartmannibacter diazotrophicus* E19T	3% NaCl (w/v)	Suarez et al. (2019)
	*dnaJ, groEL and groES*	*Klebsiella* sp. KBG6.2	24% NaCl (w/v)	Girma et al. (2020)
	*groEL, dnaK, clpXP*	*Cronobacter muytjensii* JZ38	100 mM NaCl	Eida et al. (2020)
	*dnaJ, dnaK, groES, groEL, clpB, clpC, clpE, clpX, htpX, ibpB, hsp20, hptG, hsp33*	*Bacillus megaterium* STB1	5% NaCl (w/v)	Nascimento et al. (2020)
	*smpB, hslR, ibpA, ibpB, and hspQ*	*Enterobacter roggenkampii* ED5	12% NaCl (w/v)	Guo et al. (2020)
	*grpE, yflT, dps1, hslO, groL, hfq, lepA*	*Bacillus* sp. BH32	5% NaCl (w/v)	Belaouni et al. (2022)
**Potassium transport systems**	*kdp* operon	*Klebsiella* sp. D5A	Mentioned above	Liu et al. (2016)
	*kdp operon*	*Bacillus paralicheniformis* ES-1	Mentioned above	Iqbal et al. (2022)
	*Kdp* operon*, kup, kefA, kefB/C*	*Stenotrophomonas* sp. 169	-	Ulrich et al. (2021)
	*kdp* operon*, trk, kup*	*Pantoea agglomerans* ANP8	11.6% NaCl (w/v)	Noori et al. (2021)
	*kdp* operon*, trkGA*	*Hartmannibacter diazotrophicus* E19T	Mentioned above	Suarez et al. (2019)
	*kdp* operon*, trk, kup*	*Jejubacter calystegiae*	11% NaCl (w/v)	Jiang et al. (2021)
	*kup*	*Stenotrophomonas rhizophila* IS26	7% NaCl (w/v)	Dif et al. (2022)
	*kdp* operon*, trkAGE, kup, kefBCFG, kch*	*Cronobacter muytjensii* JZ38	Mentioned above	Eida et al. (2020)
	*Kdp* operon	*Achromobacter xylosoxidans* SQU-1	100 mM NaCl	Jana and Yaish 2021
	*kdp operon, trk, kup*	*Enterobacter roggenkampii* ED5	Mentioned above	Guo et al. (2020)
	*kdp* operon	*Enterobacter* sp. SA187	1 M NaCl	Andres-Barrao et al. (2017)
	*ktrA, kefC, kdp* operon	*Bacillus* sp. BH32	Mentioned above	Belaouni et al. (2022)
	*ktrB*	*Bacillus flexus* KLBMP 4941	8% NaCl (w/v)	Wang et al. (2017)
**Na** ^**+**^ **/H** ^**+**^ **antiporters**	*nha, mrpABCDEFG*	*Hartmannibacter diazotrophicus* E19T	Mentioned above	Suarez et al. (2019)
	*Nha*	*Klebsiella* sp. D5A	Mentioned above	Liu et al. (2016)
		*Pantoea agglomerans* ANP8	Mentioned above	Noori et al. (2021)
		*Cronobacter muytjensii* JZ38	Mentioned above	Eida et al. (2020)
		*Pseudomonas thivervalensis* SC5	5% NaCl (w/v)	Nascimento et al. (2021)
		*Jejubacter calystegiae*	Mentioned above	Jiang et al. (2021)
		*Bacillus megaterium* STB1	Mentioned above	Nascimento et al. (2020)
		*Enterobacter roggenkampii* ED5	Mentioned above	Guo et al. (2020)
		*Enterobacter* sp. SA187	Mentioned above	Andres-Barrao et al. (2017)
		*Bacillus* sp. BH32	Mentioned above	Belaouni et al. (2022)
		*Achromobacter xylosoxidans* SQU-1	100 mM NaCl	Jana and Yaish 2021
	*nha, czcD*	*Pseudomonas chloritidismutans* 6L11	5% NaCl (w/v)	Zhou et al. (2022)
	*nha, mrpB, yjbQ*	*Bacillus flexus* KLBMP 4941	Mentioned above	Wang et al. (2017)
	*mrpF, nhaK*	*Stenotrophomonas rhizophila* IS26	Mentioned above	Dif et al. (2022)
**Probable ionic transporter**	*chaA, yrbG*	*Cronobacter muytjensii* JZ38	Mentioned above	Eida et al. (2020)
	*chaA, ybaL*	*Pantoea agglomerans* ANP8	Mentioned above	Noori et al. (2021)
**Na** ^**+**^ **transporters**	*natBA*	*Jejubacter calystegiae*	Mentioned above	Jiang et al. (2021)
**Na**+**, Li**+**, K**+**/H**+ **antiporter**	*mdrP*	*Bacillus* sp. BH32	Mentioned above	Belaouni et al. (2022)

The names of genera and species of bacteria and also the genes were presented in italic forms.

Incitation of K^+^ uptake is the first quick reaction to an osmotic change by bacteria (Goyal et al., 2019). Three K^+^ uptake systems have different affinities: the Kup system, which is permanently active and keeps a little level of K^+^ absorption and is not regulated through osmolality, while Trk and Kdp systems are multipartite inducible systems, which activated afterward osmotic up shock (Goyal et al., 2019). Kdp was the most common gene related to K^+^ uptake systems, which has been identified in the genomes of 55% of assessed halotolerant PGPB (Table 1).

Osmolytes are accumulated either by intake from surroundings or by *de novo* synthesis (Mishra et al., 2018). The molecular aspect of the synthesis of osmolytes is discussed in Section 4.5.

Heat-shock proteins (HSPs) or chaperones, including DnaK, DnaJ, ClpX, ClpA, ClpB, GroES, GroEL, proteases, and sHSPs, are upregulated upon osmotic stress. The main role of chaperones is to control the folding and refolding process of stress-affected proteins (Bourque et al., 2016). Clp proteins are involved in regulating ATP-dependent proteolysis. *ClpC* gene expression is induced by several stresses in *Bacillus subtilis* (Goyal et al., 2019). Furthermore, a unique osmotolerance gene *brpA* encodes a carotenoid-modifying enzyme. The product of *mazG* is nucleoside triphosphate pyrophosphohydrolase which eliminates aberrant dNTPs from damaged DNA due to stress by hydrolyzing dNTPs to pyrophosphate and dNMPs (Goyal et al., 2019). Genes affiliated with the salinity tolerance in assayed halotolerant PGPB are presented in Table 1.

The transcriptome analysis of *Chromohalobacter salexigens* ANJ207 which is a halophilic PGPB showed the expression of genes related to HSPs synthesis increased (Srivastava et al., 2022). The gene expression assay of *Azospirillum lipoferum* 4B during interaction with rice roots showed several genes related to stress response (*nhaA1*, *cspA2*, and *msrA*) and genes related to HSPs (hspD2, groES1) were induced (Drogue et al., 2014).

## 4 Biochemical and genetic mechanisms of salinity stress-alleviating bacteria in plants

**FIGURE 2:**
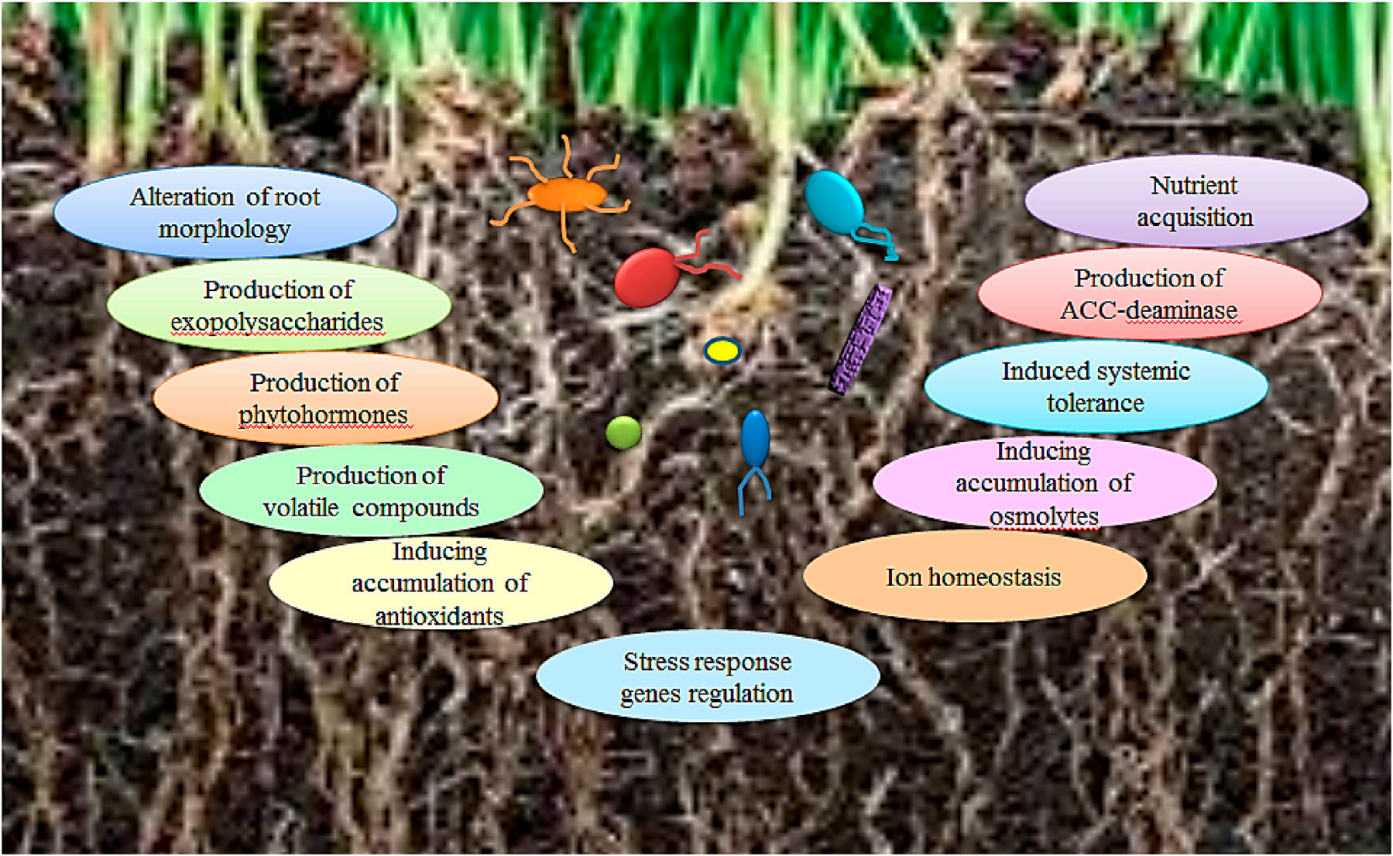
Mechanisms employed by PGPB in mitigating the salinity stress for plants.

Some PGPB show several plant-beneficial traits through the aggregation of the related genes which have been elected in these bacteria (Bruto et al., 2014). Additionally, PGPB or bacterial products offer cross-protection against other stresses due to the natural crosstalk between stress-response pathways (Rosier et al., 2018). PGPB act principally through the synthesis of numerous secondary metabolites, modulation of the transcription of several genes, and cellular communication *via* quorum sensing (Mokrani et al., 2020). PGPB colonize plants and confer salinity tolerance through alteration in root morphology, nutrient attainment, synthesis of exopolysaccharides, 1-aminocyclopropane-1-carboxylate (ACC) deaminase, phytohormones, and volatile composites, prompting accumulation of antioxidants and osmolytes, ion homeostasis, induced systemic tolerance and regulation of the stress response genes (Figure 2) (Mohammadipanah and Zamanzadeh, 2019; Etesami, 2020; Batista et al., 2021).

### 4.1 Molecular features of nutrient acquisition

The mineral nutritional estate of plants influences their capability to adjust to stresses while salinity decreases the accumulation of plant nutrients (Carmen and Roberto, 2011; Shilev, 2020). Salinity-tolerant PGPB reduce the need for chemical fertilizers through the increasing accessibility of plant nutrients (Etesami, 2020). Furthermore, PGPB improve nutrient bioavailability indirectly by increasing the root surface area (Olenska et al., 2020). Genes related to nutrient attainment in salinity stress alleviating PGPB are exhibited in Table 2.

**TABLE 2:** Genes associated with nutrient acquisition in the 20 studied genomes of PGPB alleviating osmotic stress.

Associated mechanism	Product/Role	Genes	Bacteria	References
**Nitrogen fixation**	Nitrogenase synthesis	*nifHDKMZUS WAL*	*Klebsiella* sp. D5A	Liu et al. (2016)
		*NifHDKENXBQVUSZWT*	*Hartmannibacter diazotrophicus* E19^T^	Suarez et al. (2019)
	Putative membrane complex contributing to the transfer of electron to nitrogenase	*FixABCX*		
	Nitrogen fixation protein NifU	*NifU*	*Bacillus flexus* KLBMP 4941	Wang et al. (2017)
	Nitrogen fixation protein NifU and related proteins	*IscU*	*Enterobacter roggenkampii* ED5	Guo et al. (2020)
	Nitrogen fixation protein NifU and related proteins	*iscU, fixGS*	*Pseudomonas chloritidismutans* 6L11	Zhou et al. (2022)
**Ammonia production**	Urease accessory proteins	*UreFGED*	*Klebsiella* sp. KBG6.2	Girma et al. (2020)
**Mineral phosphate solubilization**		*PqqBCDE*	*Pantoea agglomerans* ANP8	Noori et al. (2021)
		*PqqBCDEF*	*Klebsiella* sp. D5A	Liu et al. (2016)
		*PqqD*	*Klebsiella* sp. KBG6.2	Girma et al. (2020)
		*PqqBCDE*	*Hartmannibacter diazotrophicus* E19^T^	Suarez et al. (2019)
		*PqqABCDEFHI*	*Pseudomonas fluorescens* PCL1751	Cho et al. (2015)
		*PqqBCDEF*	*Cronobacter muytjensii* JZ38	Eida et al. (2020)
		*PqqBCE*	*Pseudomonas chloritidismutans* 6L11	Zhou et al. (2022)
		*pqq* operon	*Pseudomonas thivervalensis* SC5	Nascimento et al. (2021)
	Glucose dehydrogenase/GA synthesis	*Gcd*	*Cronobacter muytjensii* JZ38	Eida et al. (2020)
			*Pseudomonas fluorescens* PCL1751	Cho et al. (2015)
			*Pantoea agglomerans* ANP8	Noori et al. (2021)
			*Pseudomonas thivervalensis* SC5	Nascimento et al. (2021)
			*Klebsiella* sp. D5A	Liu et al. (2016)
			*Stenotrophomonas*sp. 169	Ulrich et al. (2021)
			*Stenotrophomonas rhizophila* IS26	Dif et al. (2022)
			*Pseudomonas chloritidismutans* 6L11	Zhou et al. (2022)
	Oxidase glucose to gluconolactone/GA synthesis	*YliI*	*Hartmannibacter diazotrophicus* E19^T^	Suarez et al. (2019)
**Organic phosphate**	C-P lyase/catalyzing organic phosphate solubilization	*phn* operon	*Hartmannibacter diazotrophicus* E19^T^	Suarez et al. (2019)
	Phytase, Exopolyphosphatase, Alkaline phosphatase	*phyC, ppx, phoD*	*Pseudomonas thivervalensis* SC5	Nascimento et al. (2021)
	Alkaline phosphatase	*phoA, phoD*	*Bacillus megaterium* STB1	Nascimento et al. (2020)
	Alkaline phosphatase, phytase, exopolyphosphatase, pyrophosphatase, polyphosphate kinase, phosphonoacetate hydrolase	*phoA, ppx, ppa ppk, phnA*	*Stenotrophomonas* sp. 169	Ulrich et al. (2021)
	Alkaline phosphatase, protein phosphatase	*alpl, cheZ*	*Stenotrophomonas rhizophila* IS26	Dif et al. (2022)
	Alkaline phosphatase	*PhoA*	*Enterobacter roggenkampii* ED5	Guo et al. (2020)
	Alkaline phosphodiesterase	*PhoD*	*Pseudomonas chloritidismutans* 6L11	Zhou et al. (2022)
	Exopolyphosphatases, pyrophosphatase	*ppx, gppA, ppa*	*Enterobacter* sp. SA187	Andres-Barrao et al. (2017)
**Sulphur acquisition**	Sulfonate and sulfate transport	*ssuABC* and *cysPUWA*	*Pantoea agglomerans* P5	Shariati et al. (2017)
	Sulfate reduction pathway, and sulfate transport	*cysPUWA, ylnA, sulP*	*Bacillus megaterium* STB1	Nascimento et al. (2020)
	Sulfonate transport and degradation genes	*SsuABCD*	*Bacillus megaterium* STB1	Nascimento et al. (2020)
	Sulfate reduction pathway, and sulfate transport	*cysPTWADNCQH* and *sir*	*Hartmannibacter diazotrophicus* E19^T^	Suarez et al. (2019)
	Sulfonate transport and degradation genes Taurine transport	*ssuABCE tauABC*	*Hartmannibacter diazotrophicus* E19^T^	Suarez et al. (2019)
	Sulfate reduction pathway, and sulfate transport taurine transport and metabolism transport and metabolism of alkanesulfonate metabolism of thiosulfate	*cysPUWANDCHJI tauACBD ssuACBDE glpE*	*Cronobacter muytjensii* JZ38	Eida et al. (2020)
	Sulfate reduction pathway, and sulfate transport taurine transport and metabolism degradation of sulfones and alkanesulfonates	*Cis, sulP, cysPUWA, tau, sfnG, ssuABCD*	*Pseudomonas thivervalensis* SC5	Nascimento et al. (2021)
**Acquisition of Iron**	Enterobactin synthesis/siderophore	*EntABCDEF*	*Cronobacter muytjensii* JZ38	Eida et al. (2020)
		*EntABCDEF*	*Klebsiella* sp. D5A	Liu et al. (2016)
		*entABCDEF, fes, fepA*	*Enterobacter* sp. SA187	Andres-Barrao et al. (2017)
		*fes, entFSD, fepA*	*Enterobacter roggenkampii* ED5	Guo et al. (2020)
		*1* gene	*Brevibacterium sediminis* MG-1	Lutfullin et al. (2022)
	Aerobactin synthesis/siderophore	*IucABCD*	*Cronobacter muytjensii* JZ38	Eida et al. (2020)
		*4* gene	*Brevibacterium sediminis* MG-1	Lutfullin et al. (2022)
		*iucA/iucC* family gene cluster	*Pseudomonas chloritidismutans* 6L11	Zhou et al. (2022)
	Pyoverdine synthesis/siderophore	*PvdAEFGHIJMNOPQS*	*Pseudomonas fluorescens* PCL1751	Cho et al. (2015)
		*Pvd*	*Pseudomonas thivervalensis* SC5	Nascimento et al. (2021)
		*PvdAQSLHGDJEONMP*	*Pseudomonas* sp. UW4	Duan et al. (2013)
	Histocorrugatin synthesis/siderophore	*HcsABCDEFGHIJKL*	*Pseudomonas thivervalensis* SC5	Nascimento et al. (2021)
	Pyochelin synthesis/siderophore	*PchABCDHIKPR*	*Pseudomonas fluorescens* PCL1751	Cho et al. (2015)
	Siderophore synthesis	*RhaABCDEF*	*Bacillus megaterium* STB1	Nascimento et al. (2020)
	Rhizobactin siderophore biosynthesis	*RhbCDEF*	*Bacillus flexus* KLBMP 4941	Wang et al. (2017)
	Petrobactin siderophore biosynthesis	*4 gene*	*Brevibacterium sediminis* MG-1	Lutfullin et al. (2022)

The names of genera and species of bacteria and also the genes were presented in italic forms.

**Nitrogen** content of saline land is insufficient for normal plant growth (Kapoor and Kanwar, 2019). Bacteria can convert the non-available nitrogen to more available forms through mineralization, nitrification, and fixation. Mineralization entails a flow of microbial and enzymatic actions that transform soil organic nitrogen to inorganic (Olenska et al., 2020). PGPB can fix atmospheric nitrogen into ammonia form through nitrogen fixation (Vaishnav et al., 2016). Nitrogen can be converted naturally into ammonia by lightning or fires but is mainly biologically fixed by diazotrophs. Diazotrophs produce ammonia using nitrogenase encoded by *nif* genes located on chromosomes or plasmids like in most *Rhizobium* (Glick et al., 1999b; Olenska et al., 2020). The *nif* genes are organized in a single cluster along with seven discrete operons encode 20 diverse proteins (Glick, 2012). *Nif* genes contain regulatory genes (*nifLA*), structural genes (*nifHDK*), and supplementary genes (as *nifBEMNQSUVW*) (Glick et al., 1999b; Tomer et al., 2016). *NifH* gene can be applied as a valuable marker to illustrate the diazotrophs (Tomer et al., 2016). Among 20 assessed halotolerant PGPB, the *nif* gene cluster has been detected in the genomes of *Klebsiella* sp. D5A and *Hartmannibacter diazotrophicus* E19T (Table 2).

The transcriptome analysis of *Paenibacillus* sp. Strain S02 in interaction with barely showed the expression of the *nif* operon was upregulated (Li et al., 2021).

The metatranscriptome study of a group of different PGPB (*Rhizobium phaseoli*, *Sinorhizobium americanum*, *Azospirillum brasilense*, *Bacillus subtillis,* and *Methylobacterium extorquens*) that were inoculated to maize showed that the expression of *nif* genes of *Azospirillum* increased and it was the cause of nitrogen fixation in maize (Gomez-Godinez et al., 2018).

Numerous studies have shown PGPB-priming changes the expression of genes (Fadiji et al., 2022). A new non-coding RNA (ncRNA) was known in *Pseudomonas stutzeri* (NfiS), which controls the *nif* genes expression and presents on the core genome. NfiS acts through post-transcriptional modulation of dinitrogenase *nifK* mRNA and induces the RpoN/NtrC/NifA regulatory cascade, which is an activator of transcription of every *nif* operons by unknown procedures (Olenska et al., 2020).

In addition, some of the PGPB affect the root nutrient transport systems. *Bacillus* spp. trigger the expression of genes connected to nitrate and ammonium intake and transfer in *Arabidopsis thaliana* (Olenska et al., 2020).

**Sulfur,** as the secondary essential macronutrient, is only accessible for plants in the form of sulfate (5% of total soil S) (Vaishnav et al., 2016). Alkanesulfonates as the main ingredients of organosulfur mixtures in agricultural lands, are transmitted inside the cell using aliphatic sulfonate ABC transport (*ssuABC*) and transformed into sulfite by the alkanesulfonate monooxygenase (*ssuD*) and an NADPH-dependent FMN reductase (*ssuE*) (Suarez et al., 2019). There are multiple copies of the *ssuABC* genes on the *Hartmannibacter diazotrophicus* E19T genome. Remarkably, plasmid HDIAp1 encloses additional CDSs of *ssuABC* and *ssuE*. Furthermore, the genome of E19T carries the *tauABC* genes related to taurine transfer into the bacterial cell for higher decomposition of taurine to alanine and sulfoacetaldehyde using the taurinepyruvate aminotransferase (Tpa) (Suarez et al., 2019).

**Potassium** (K) is the third primary essential nutrient used by plants (Kumar et al., 2020). Nevertheless, most of the K is not accessible for plant absorption. Furthermore, salinity stress reduces K availability to plants. In this condition, K-solubilizing bacteria (KSB) are needed for plant survival (Vaishnav et al., 2016; Kumar et al., 2020). KSB solubilize K through chelation, acidolysis, synthesizing organic and inorganic acids, polysaccharides, and exchange reactions (Etesami et al., 2017).

**Iron,** as the fourth plentiful element utilized by the majority of living organisms is applied by almost 140 enzymes as a cofactor. In saline land, the solubility of ferric is lessened because of increased pH. Siderophores are iron-chelating means produced by most PGPB with a vast chemical diversity including peptidic, aminoalkane, and citric acid-based siderophores (Vaishnav et al., 2016). Siderophores are commonly made *via* non-ribosomal peptide synthetases (NRPSs) or polyketide synthase, which collaborates with NRPS modules (Olenska et al., 2020). The genes related to enterobactin (*ent*) and pyoverdine (*pvd*) synthesis have been identified with more prevalence in the genomes of assessed halotolerant PGPB (Table 2). The genes of enterobactin and pyoverdine synthesis were the most common identified genes related to siderophores synthesis in assessed bacteria belonging to Enterobacteriaceae (67%) and Pseudomonadaceae (75%) families, respectively.

The transcriptome analysis of *Paenibacillus* sp. in interaction with barely showed the expression of the siderophore cluster was upregulated (Li et al., 2021).

**Phosphorus** is a vital macronutrient for plants. Only around 4% of the phosphorus in soil is accessible to plants (Aslam et al., 2020; Olenska et al., 2020). Phosphate-solubilizing bacteria (PSB) hydrolyze unavailable forms of phosphorus into available forms by several mechanisms (Leontidou et al., 2020). Bacteria can do phosphate solubilization through releasing of H^+^ to the outer surface in interchange for cation intake, but phosphates are released mainly by soil acidification through organic acids. Bacterial organic acids are produced due to direct oxidation in the periplasmic space (Olenska et al., 2020). Organic acids chelate phosphate binding cations, causing a decrease in pH and providing phosphate anions (Olenska et al., 2020). The most prevalent organic acids consist of gluconic acid (GA) and 2-ketogluconic acid (Olenska et al., 2020). Glucose-1-dehydrogenase (*gcd*) synthesizes GA, and pyrrolo-quinolone quinine (PQQ) acts as its co-factor. Furthermore, GA dehydrogenase (*gad*) plays a role in GA production and its conversion to 2-ketogluconate (Shariati et al., 2017). Additionally, hydrogen sulfide (H_2_S) plays a role in the solubilization of phosphate. H_2_S, in reaction with ferric phosphate, produces ferrous sulfate and releases phosphate (Shariati et al., 2017). P-organic substrates enzymatically hydrolyze into inorganic kinds by PGPB (Khatoon et al., 2020). These enzymes are non-specific acid phosphatases (NSAPs), including phytases, acid and alkaline phosphomonoesterases (phosphatases), phosphonatases, and C-P lyases (Suarez et al., 2019; Olenska et al., 2020). The genes related to mineral phosphate solubilization (*pqq* and *gcd*) and organic phosphate solubilization have been identified in the genomes of 11 of assessed halotolerant PGPB (Table 2).

### 4.2 Bacterial phytohormones and modulation of their expression in salinity condition

Phytohormones protect plants against abiotic stresses, and PGPB can modulate the level of endogenous phytohormones (Mohammadipanah and Zamanzadeh, 2019; Khan et al., 2020a). PGPB produce analogs of phytohormones, metabolize them, or influence the synthesis of plant hormone and signal transduction (Tsukanova et al., 2017; Olenska et al., 2020). PGPB hormones can trigger the division and growth of plant cells, alter root characteristics and play a significant role in organizing an array of genes, their regulators, and several signal transduction pathways when plants are exposed to abiotic stresses and make crops tolerant to the stresses (Mohammadipanah and Zamanzadeh, 2019; Etesami, 2020). The main phytohormones are abscisic acid, gibberellins, auxins, ethylene, salicylic acid, and cytokinins (Olenska et al., 2020). Genes of phytohormones synthesis in salinity stress mitigating PGPB are presented in Table 3.

**TABLE 3:** Identified genes related to hormone synthesis in the 20 studied genomes of saline soil PGPB.

Associated mechanism	Product/Role	Genes	Bacteria	References
**IAA production**	Aminotransferase, pyruvate decarboxylase and numerous phenolic acid decarboxylase, aldehyde dehydrogenase, indole-3-pyruvic acid (IPyA) pathway/amidase, indole-3-acetamide (IAM) pathway	*patB*, *yclB* homolog, *dhaS, ami*	*Bacillus megaterium* STB1	Nascimento et al. (2020)
	Indole–3–pyruvate decarboxylase/indole-3-pyruvic acid (IPyA) pathway. Indoleacetamide hydrolase, Tryptophan 2-monooxygenase, indole-3-acetamide (IAM) pathway	*ipdC iaaH, homologous gene to iaaM*	*Pantoea agglomerans* ANP8	Noori et al. (2021)
	Indole–3–pyruvate decarboxylase (indole-3-pyruvic acid (IPyA) pathway)	*ipdC*	*Bacillus* sp. BH32	Belaouni et al. (2022)
	Amidase (IAM pathway)/Aldehyde dehydrogenase, indole pyruvate ferredoxin oxidoreductase (IPyA)	*amiE, dhaS*	*Stenotrophomonas* sp. 169	Ulrich et al. (2021)
	Indole–3–pyruvate decarboxylase, Aldehyde dehydrogenase (indole-3-pyruvic acid (IPyA) pathway)/Amidase (IAM pathway)	*ipdC, aldHT amiE*	*Stenotrophomonas rhizophila* IS26	Dif et al. (2022)
	indole-3-pyruvic acid (IPyA pathway)	*aspC, ipdC, aldA, aldB*	*Cronobacter muytjensii* JZ38	Eida et al. (2020)
	Tryptophan 2-monooxygenase, amidase (IAM pathway)	*iaaM* homolog*, amiE, yafV*	*Pseudomonas thivervalensis* SC5	Nascimento et al. (2021)
	Indole-3-acetonitrile (IAN) and Indole-3-pyruvate (IPyA) pathways	*nthA, nthB, amiE, ipdC*	*Klebsiella* sp. D5A	Liu et al. (2016)
	Indole-3-acetamide (IAM) and indole-3-acetonitrile (IAN) pathways	*nthA, nthB, IaaM, amiE*	*Pseudomonas* sp. UW4	Duan et al. (2013)
	Indole-3-pyruvic acid (IPyA pathway)	*aspC, aldA, aldB*	*Enterobacter* sp. SA187	Andres-Barrao et al. (2017)
	Amidase (IAM pathway)/indole-3-pyruvate. Monooxygenase (IPyA pathway)	*amiE, YUC9*	*Pseudomonas chloritidismutans* 6L11	Zhou et al. (2022)
	Tryptophan biosynthetic pathway	*trpABCDE*	*Cronobacter muytjensii* JZ38	Eida et al. (2020)
		*trpEG*, *trpD*, *trpF*, *trpC*, *trpAB*	*Stenotrophomonas* sp. 169	Ulrich et al. (2021)
		*trpCF, trpS, trpE, trpB, trpGD*	*Enterobacter roggenkampii* ED5	Guo et al. (2020)
		*trpA, B, C, E, S, R, and GD*	*Pantoea agglomerans* ANP8	Noori et al. (2021)
		*trpABFD*	*Bacillus* sp. BH32	Belaouni et al. (2022)
	Tryptophan synthase	*trpAB*	*Stenotrophomonas rhizophila* IS26	Dif et al. (2022)
		*trpAB*	*Bacillus megaterium* STB1	Nascimento et al. (2020)
**Cytokinin synthesis**	tRNA dimethylallyltransferase and tRNA-2-methylthio-N6 dimethylallyladenosine synthase, cytokinin riboside 50-monophosphate phosphoribohydrolase	*miaA, miaB, yvdD*	*Bacillus megaterium* STB1	Nascimento et al. (2020)
	tRNA dimethylallyltransferase and tRNA-2-methylthio-N6 dimethylallyladenosine synthase	*miaA, miaB, and miaE*	*Pseudomonas thivervalensis* SC5	Nascimento et al. (2021)

The names of genera and species of bacteria and also the genes were presented in italic forms.

**Abscisic acid (ABA)** is recognized as a stress hormone and is upregulated in salinity stress (Kumar et al., 2020). ABA assists in the accumulation of osmolytes, Ca^2+^ and K^+^, modulates cell ion balance, prompts the roots elongation and the appearance of lateral roots, increases old leaf shedding, and controls leaf stomatal closure (Bhat et al., 2020; Kumar et al., 2020; Nian et al., 2021). ABA has two principal functions, regulates metabolism and transfer of itself through posttranslational modulation, and also it interacts with core transcription factors which are modulated through ABA and other phytohormones. The amount of ABA increases under osmotic stress through elevated expression of multiple genes of ABA production, including genes of aldehyde oxidase, zeaxanthin epoxidase, 9-cis-epoxycarotenoid dioxygenase, and molybdenum cofactor sulturase (Khan et al., 2020a). Several PGPB strains can synthesize and degrade the ABA (Tsukanova et al., 2017). Nevertheless, the production of ABA *via* bacteria or the excitation of ABA synthesis in plants has been scarcely studied (Gowtham et al., 2020).

**Auxins** are involved in nearly all aspects of plant physiology and the mitigation of abiotic stress (Olenska et al., 2020). Bacteria affect plant auxin homeostasis by synthesizing auxin, affecting the expression of the plant auxin production genes, and transport or signaling machinery (Tsukanova et al., 2017). Auxins are synthesized and excreted by above 80% of the rhizobacteria (Olenska et al., 2020). In bacteria, five of the six pathways for auxin production depend on tryptophan. These pathways have been categorized according to their intermediate including indole-3-pyruvate (IPyA), indole-3-acetamide (IAM), indole-3-acetonitrile, tryptophan side-chain oxidase, tryptamine, and tryptophan independent (Gamalero and Glick, 2011). IAM and IPyA are apparently two main microbial pathways (Spaepen, 2015; Gupta et al., 2016). At the IAM pathway, tryptophan is first transformed to IAM through tryptophan monooxygenase. Then, IAA is synthesized from IAM through an IAM hydrolase (Spaepen, 2015). Most PGPB apply the IPyA pathway (Gupta et al., 2016; Khatoon et al., 2020). In the IPyA pathway, tryptophan is transaminated to IPyA *via* a pyridoxal phosphate-dependent aromatic aminotransferase. Then IPyA decarboxylase (IPDC, *ipdC* gene) transforms IPyA to indole-3-acetaldehyde (IAAld). Lastly, IAAld is transformed into IAA (Glick et al., 1999a; Olenska et al., 2020). Multiple IAA biosynthetic pathways can be existent and active in one single organism as there are both the IAM and the IPyA pathway in the *Pantoea agglomerans* genome (Spaepen, 2015). The genes related to IAA biosynthesis have been identified in the genomes of more than 70% of assessed halotolerant PGPB and the genes related to IPyA and IAM pathways were more prevalent (80% and 60%, respectively). Over 63% of assessed IAA producing bacteria contain more than one pathway (Table 3).

The transcriptomic analysis of *Paenibacillus polymyxa* YC0136 in intraction with tobacco showed the expression of the *ilvB* gene in strain YC0136 was up-regulated. *IlvB* encodes aldehyde dehydrogenase, which transforms indole-3-acetaldehyde into IAA (Liu et al., 2020).

The expression of genes related to indole-3-acetic acid (IAA) synthesis including the genes of nitrile hydrolase, IAM hydrolase, and aldehyde dehydrogenase were found in transcriptome assay of *Burkholderia phytofirmans* PsJN colonizing potato under drought stress (Sheibani-Tezerji et al., 2015).

**FIGURE 3:**
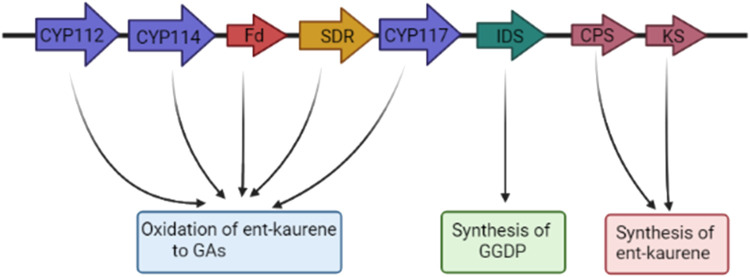
Bacterial gibberellin synthetic core operon. Isoprenyl diphosphate synthase (IDS) synthesizes geranylgeranyl diphosphate (GGDP), the pair of diterpene cyclases (copalyl diphosphate (CDP) synthase (CPS) and ent-kaurene synthase (KS)) catalyze the synthesis of ent-kaurene from GGDP. Ent-kaurene is converted to GAs through several oxidation steps by a minimum of three cytochromes P450 (CYPs), CYP112, CYP114, and CYP117, short-chain alcohol dehydrogenase/reductase (SDR), and a ferredoxin (Fd) (created in BioRender.com).

**Gibberellins (GAs)** are tetracyclic diterpenoid carboxylic acid derivatives that can alleviate the abiotic stress (Khatoon et al., 2020). PGPB affect the quantity of gibberellin in plants in a manner similar to auxin. Gibberellin-producing PGPB conserve plants from stress by modulating antioxidant levels, stimulating the absorption of calcium ions and other nutrients (Khatoon et al., 2020). Among 136 known GA structures, GA3 is most often synthesized by bacteria (Olenska et al., 2020). In recent years, the GA biosynthesis pathway in bacteria has been clarified (Salazar-Cerezoa et al., 2018). Bacterial synthesis is related to a cytochrome P450-rich operon which contains a cluster of core genes coding for eight enzymes (Figure 3). Numerous copies of the operon further than these core genes enclose an isopentenyl diphosphate isomerase (IDI) and CYP115. GA operon is typically located on plasmids, though; the basis of this genetic transfer has not been clarified (Nagel et al., 2018; Olenska et al., 2020). Some bacteria, such as *Burkholderia phytofirmans* PsJN don’t produce gibberellins but positively regulate the expression of the related genes in the synthesis of gibberellin in *A. thaliana* (Tsukanova et al., 2017).

**Cytokinins** are adenine derivatives involved in root differentiation, shoot formation, and stress alleviation (Numan et al., 2018). Many PGPB can synthesize cytokinin, which is started *via* isopentenyl transferase (*ipt* gene) that catalyzes the transportation of the isopentenyl moiety from dimethylallyl diphosphate to adenosine monophosphate. The influence of cytokinin in the case of pathogens is inhibitory on plants, and is stimulatory in the case of PGPB as the amounts of the synthesized cytokinin by PGPB are less than those from phytopathogens (Shilev, 2020).

**Ethylene** acts in several stages of plant ontogenesis and stress signaling pathways (Kumar et al., 2020). The ethylene synthesis is under tight control at transcriptional and post-transcriptional levels (Choudhary et al., 2015). The upper concentration of ethylene accumulation under stress condition can prevent plant growth and accelerate senescence (Olenska et al., 2020). Ethylene is produced through ACC oxidase (ACCO) from ACC (Olenska et al., 2020; Shilev, 2020). When ACCO synthesizes ethylene higher than its threshold amount can cause “stress ethylene” in the plant (Olanrewaju et al., 2017). The expression and activity of ACC synthase (ACCS) are increased by IAA increase in plants (Glick et al., 1999a; Olenska et al., 2020). ACC is exuded into the soil surrounding roots and is reabsorbed *via* the roots (Martinez-Viveros et al., 2010). PGPB mediate ethylene homeostasis by decreasing its amount within plants due to their rhizobitoxine and ACC deaminase enzyme (ACCD) generation (Olenska et al., 2020). Rhizobitoxine is an enol-ether amino acid and acts as a competitive inhibitor of ACCS and the production of ACCD. The activities of rhizobitoxine relate to the inhibition of β-cystathionase in the methionine (precursor of ethylene) synthesis and ACCS in the ethylene production pathway. PGPB decrease the deleterious effect of ethylene by eliminating ACC (Choudhary et al., 2015). PGPB may also affect plant ethylene concentration by influencing the expression of the genes connected to ethylene production; genes of ACCS and ACCO enzymes. For example, *Burkholderia phytofirmans* PsJN upregulates ACCS and ACCO genes in *A. thaliana* (Tsukanova et al., 2017).

Salicylic acid (SA) is a main phytohormone that modulates different aspects of plant growth, and biotic and abiotic stress responses. In bacteria, enzymes related to SA biosynthesis are encoded by the NRPS gene cluster. In first step which is common in both bacteria and plants, chorismate is converted to isochorismate by isochorismate synthase, and then SA is produced from isochorismate using isochorismate pyruvate lyase (Chen et al., 2019; Mishra and Baek, 2021).The metabolites related to the auxins and zeatins biosynthesis pathways were explored from three PGPB (*Pseudomonas putida* IDE-01, *Azospirillum brasilense* IDE-06, and *Bacilus megaterium* IDE-14) in interaction with maize and rice (Garcia et al., 2022).

### 4.3 Molecular aspects of resistance induced by ACC deaminase (ACCD)

Environmental stresses lead to increased ethylene production in the plant which hampers the growth of plants (Kumari et al., 2016). The ACCD-synthesizing bacteria can diminish the deleterious impact of the different stresses on plants by catabolizing ACC to α -ketobutyrate (precursor of leucine) and ammonia, leading to the low expression of the ACCO gene (Mohammadipanah and Zamanzadeh, 2019; Fadiji et al., 2022). ACCD is not secreted by bacteria, and ACC is secreted from roots and is then absorbed *via* the PGPB carrying ACCD (Kumari et al., 2016). ACCD is a cytoplasmic enzyme encoded by the *acdS* gene and is highly controlled according to the existence or lack of oxygen, amount of ACC, and aggregation of the product (Kumari et al., 2016; Jaya et al., 2019).

**FIGURE 4:**
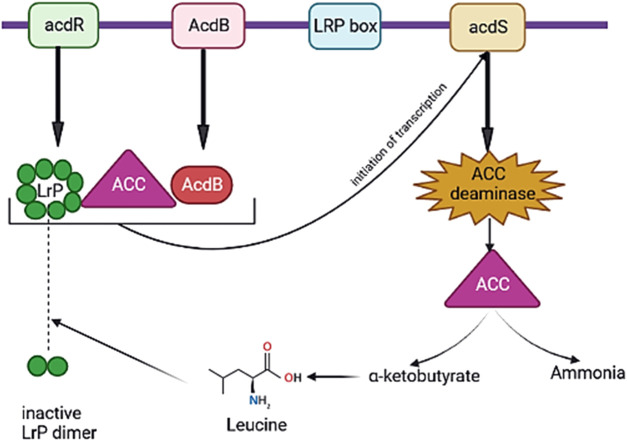
Regulation of expression of acdS (ACC deaminase synthase) gene through the leucine-responsive regulatory protein (LrP) and AcdB protein. When 1-aminocyclopropane-1-carboxylic acid (ACC) exists, active octamer of LrP is encoded through *acdR* gene. As a result of the interaction of active LrP with AcdB protein and ACC, a complex is generated, which starts the transcription of *acdS* gene, and ACCD is synthesized. The interaction of leucine as the amount of it rises in the bacteria with the active LrP octamer makes an inactive LrP dimer that causes stopping the transcription of the *acdS* gene (created in BioRender.com).

Many of the *acdS* genes have been principally modulated through the leucine-responsive regulatory protein (LrP) and AcdB protein. LrP is encoded by the ACCD regulatory (*acdR*) gene located upstream of the *acdS* gene (Figure 4) (Kumari et al., 2016). In addition, *acdS* gene expression is controlled through various regulatory proteins in diverse bacterial species for example, σ70 and LrP in *Burkholderia* sp. CCGE 1002 and *Burkholderia phymatum* STM 815, *nifA1*, *nifA2*, and σ54 in *Mesorhizobium loti* (Kumari et al., 2016). Moreover, some of the PGPB synthesize homologs of ACCD, d-cysteine desulfhydrase encoded *via dcyD* (Suarez et al., 2019). The potential genes for ACCD activity of *Hartmannibacter diazotrophicus* E19T are suggested as *ygeX,* and *tdcB* as their products have ammonia-lyase activity with pyridoxal phosphate dependencies like genes *acdS* and *dcyD* (Suarez et al., 2019) (Table 4).

**TABLE 4:** Genes related to 1-aminocyclopropane-1-carboxylate (ACC) deaminase in the genomes of 20 assessed halotolerant PGPB.

Associated mechanism	Product/Function	Genes	Bacteria	References
**ACC deaminase**	1-aminocyclopropane-1-carboxylate (ACC) deaminase	*acdS*	*Pseudomonas fluorescens* PCL1751	Cho et al. (2015)
			*Pseudomonas thivervalensis* SC5	Nascimento et al. (2021)
			*Pseudomonas* sp. UW4	Duan et al. (2013)
**Homologs of ACC deaminase**	l-threonine dehydratase, diaminopropionate ammonia-lyase	*tdcB, ygeX*	*Hartmannibacter diazotrophicus* E19^T^	Suarez et al. (2019)
**ACC deaminase**	1-aminocyclopropane-1-carboxylate (ACC) deaminase, d-cysteine desulfhydrase	*acdS, dcyD*	*Achromobacter xylosoxidans* SQU-1	Jana and Yaish 2021
	d-cysteine desulfhydrase	*dcyD*	*Enterobacter roggenkampii* ED5	Guo et al. (2020)
	ACC deaminase	*rimM*	*Bacillus paralicheniformis* ES-1	Iqbal et al. (2022)
	Pyridoxal phosphatedependent deaminase	*cuyA*	*Bacillus* sp. BH32	Belaouni et al. (2022)
	ACC deaminase	ACC deaminase synthesis gene	*Pseudomonas chloritidismutans* 6L11	Zhou et al. (2022)

### 4.4 Molecular basis of induced systemic tolerance

Induced systemic tolerance is presented as physical and chemical changes triggered by PGPB and causes enhanced tolerance of plants to abiotic stress (Sharma et al., 2016). It has been proposed that adaptation to stresses is related to pre-existing or “memory” defenses. “Memory” defenses in plants are induced *via* priming with specified chemicals and microbes. The percept of plants to chemicals with external origin and microbe-associated molecular patterns (MAMPs) can prompt reaction *versus* abiotic stresses (Vaishnav et al., 2016). PGPB produce diverse signaling molecules comprising cyclodipeptides, cytokinins, volatile organic compounds (VOCs), quorum sensing molecules, antioxidants, and ACCD, which triggers systemic resistance in plants (Arora et al., 2020; Etesami, 2020).

### 4.5 Molecular basis of osmolytes accumulation

The biosynthesis of osmolytes (compatible solutes) by PGPB and plants in reaction to stress operates in synergism to improve plant growth (Kaushal and Wani, 2016). Osmolytes include proline, quaternary ammonium compounds, sugars, betaines, polyamines, polyhydric alcohols, other amino acids, and water-stress proteins such as dehydrins (Vurukonda et al., 2016). Synthesis of osmolytes *via* PGPB is almost certainly faster than their related host plants, and plants prefer the uptake of osmolytes (Ilangumaran and Smith, 2017; Sekar et al., 2019).

**Proline** is a significant osmolyte having a multifunctional role, including maintenance of cytosolic pH, antioxidant activity, and function as molecular chaperone during osmotic stresses (Carmen and Roberto, 2011; Kaushal and Wani, 2016). Plants inoculated with bacteria illustrate a higher amount of proline but whether it is because of upregulation of the proline biosynthesis pathway or is absorbed from the rhizosphere has not been elucidated (Kaushal and Wani, 2016). In most of the bacteria, biosynthesis of proline implicates the united function of γ-glutamyl kinase, γ-glutamyl phosphate reductase, and 1-pyrroline-5-carboxylate reductase enzymes, which are encoded by *proB*, *proA*, and *proC* genes, respectively (Sunita et al., 2020). Nitrogen-fixing bacteria show elevated proline metabolism because of the enhanced acting of proline dehydrogenase (PDH) under the salinity stress condition (Sunita et al., 2020).

**Glycine betaine** (GB) (N,N,N-trimethyl glycine) is a quaternary ammonium synthesized by choline oxidase, aiding plants to tolerate stress through the stability of membranes, proteins, and the action of RuBisCO (Das et al., 2015; Kaushal and Wani, 2016). *Bacillus subtilis* can produce GB by oxidizing the choline to glycine betaine aldehyde through type III alcohol dehydrogenase, and finally, a glycine betaine aldehyde dehydrogenase synthesizes GB (Sunita et al., 2020). In *Arthrobacter globiformis,* the *codA* gene encodes choline oxidase (Kumar et al., 2020). More than 50% of assessed halotolerant PGPB contain the genes related to glycine betaine synthesis (Table 5).

**TABLE 5:** Identified genes related to osmolyte production in the 20 studied genomes of halotolerant PGPB.

Osmoprotectants	Product/Function	Genes	Bacteria	References
putrescine and spermidine synthesis	Arginine decarboxylase, agmatinase, and spermidine synthase	*speABE*	*Klebsiella* sp. D5A	Liu et al. (2016)
	Arginine decarboxylase, agmatinase, and spermidine synthase	*speABE*	*Enterobacter* sp. SA187	Andres-Barrao et al. (2017)
	Arginine decarboxylase, Agmatinase, S-adenosylmethionine decarboxylase, and spermidine synthase	*speABDE*	*Bacillus megaterium* STB1	Nascimento et al. (2020)
	Arginine decarboxylase, agmatinase, S-adenosylmethionine decarboxylase, and spermidine synthase	*speABHE*	*Bacillus flexus* KLBMP 4941	Wang et al. (2017)
	S-adenosylmethionine decarboxylase, spermidine synthase, Spermidine/spermine N (1)-acetyltransferase	*speDEG*	*Stenotrophomonas rhizophila* IS26	Dif et al. (2022)
	Arginine decarboxylase, Spermidine N (1)-acetyltransferase, spermidine synthase	*speAGE*	*Bacillus paralicheniformis* ES-1	Iqbal et al. (2022)
	Arginine decarboxylase, ornithine decarboxylase agmatinase, spermidine synthase, and S-adenosylmethionine decarboxylase	*speACBED*	*Cronobacter muytjensii* JZ38	Eida et al. (2020)
	Arginine decarboxylase, agmatine deiminase, N-carbamoyl putrescine amidase, spermidine synthase, S-adenosylmethionine decarboxylase, methionine adenosyltransferase	*speA, aguA, aguB, speE, speD, metK*	*Stenotrophomonas* sp. 169	Ulrich et al. (2021)
Putrescine synthesis	Arginine decarboxylase, Agmatine deiminase, Putrescine carbamoyltransferase, ornithine decarboxylase	*speA, aguA, aguB, and speC*	*Pseudomonas thivervalensis* SC5	Nascimento et al. (2021)
Trehalose synthesis	trehalose phosphorylase, treP pathway	*treP*	*Bacillus paralicheniformis* ES-1	Iqbal et al. (2022)
	Trehalose synthase, treS pathway/Trehalose-6-phosphate synthase and trehalose-6-phosphate phosphatase, otsA/otsB pathway	*treS, otsAB*	*Klebsiella* sp. D5A	Liu et al. (2016)
	Trehalose-6-phosphate synthase and trehalose-6-phosphate phosphatase, otsA/otsB pathway	*otsAB*	*Hartmannibacter diazotrophicus* E19T	Suarez et al. (2019)
	Trehalose synthase, treS pathway/maltooligosyltrehalose synthase/hydrolase/treY/treZ pathway	*treS treY, and treZ*	*Pseudomonas fluorescens* PCL1751	Cho et al. (2015)
	otsA/otsB pathway treY/treZ pathway	*otsAB treY, and treZ*	*Cronobacter muytjensii* JZ38	Eida et al. (2020)
	Alpha -trehalose-phosphate synthase, trehalose-phosphatase, otsA/otsB pathway/malto-oligosyltrehalose synthase, malto-oligosyltrehalose trehalohydrolase, treY/treZ pathway	*otsAB treY, and treZ*	*Stenotrophomonas* sp. 169	Ulrich et al. (2021)
	Alpha -trehalose-phosphate synthase, trehalose-phosphatase, otsA/otsB pathway	*otsAB*	*Stenotrophomonas rhizophila* IS26	Dif et al. (2022)
	Alpha -trehalose-phosphate synthase, trehalose-phosphatase, otsA/otsB pathway/malto-oligosyltrehalose synthase, malto-oligosyltrehalose trehalohydrolase, treY/treZ pathway/Trehalose synthase, treS pathway	*otsAB treY, treZ treS*	*Pseudomonas chloritidismutans* 6L11	Zhou et al. (2022)
	Trehalose synthase, treS pathway/maltooligosyltrehalose synthase, hydrolase, treY/treZ pathway	*treS, treYZ*	*Pseudomonas thivervalensis* SC5	Nascimento et al. (2021)
	Trehalose synthase, treS pathway/maltooligosyltrehalose synthase, hydrolase, treY/treZ pathway	*treS, treYZ*	*Pseudomonas* sp. UW4	Duan et al. (2013)
	Trehalose 6-phosphate synthase and trehalose 6-phosphate phosphatase, otsA/otsB pathway/malto-oligosyltrehalose trehalohydrolase, treZ pathway	*otsAB treZ*	*Enterobacter* sp. SA187	Andres-Barrao et al. (2017)
Glycine-betaine synthesis	Choline dehydrogenase and betaine aldehyde dehydrogenase	*betAB*	*Cronobacter muytjensii* JZ38	Eida et al. (2020)
			*Klebsiella* sp. D5A	Liu et al. (2016)
			*Hartmannibacter diazotrophicus* E19T	Suarez et al. (2019)
			*Stenotrophomonas* sp. 169	Ulrich et al. (2021)
			*Stenotrophomonas rhizophila* IS26	Dif et al. (2022)
			*Bacillus paralicheniformis* ES-1	Iqbal et al. (2022)
			*Pseudomonas chloritidismutans* 6L11	Zhou et al. (2022)
	Choline dehydrogenase and betaine aldehyde dehydrogenase	*betABC*	*Pseudomonas thivervalensis* SC5	Nascimento et al. (2021)
	Betaine aldehyde dehydrogenase	*betB*	*Bacillus megaterium* STB1	Nascimento et al. (2020)
	Betaine aldehyde dehydrogenase	*gbsA*	*Pseudomonas fluorescens* PCL1751	Cho et al. (2015)
Ectoine synthesis	Ectoine synthase	*ectC*	*Jejubacter calystegiae*	Jiang et al. (2021)
	Diaminobutyric acid aminotransferase, diaminobutyric acid acetyltransferase, and ectoine synthase	*ectB, ectA, and ectC*	*Brevibacterium sediminis* MG-1	Lutfullin et al. (2022)
	Diaminobutyric acid aminotransferase, diaminobutyric acid acetyltransferase, and ectoine synthase	*ectB, ectA, and ectC*	*Pseudomonas chloritidismutans* 6L11	Zhou et al. (2022)
Proline synthesis	γ-glutamyl kinase, γ-glutamyl phosphate reductase, and 1-pyrroline-5-carboxylate reductase	*proB, proA,* and *proC*	*Hartmannibacter diazotrophicus* E19T	Suarez et al. (2019)
			*Cronobacter muytjensii* JZ38	Eida et al. (2020)
			*Pseudomonas thivervalensis* SC5	Nascimento et al. (2021)
			*Bacillus megaterium* STB1	Nascimento et al. (2020)
			*Stenotrophomonas* sp. 169	Ulrich et al. (2021)
			*Stenotrophomonas rhizophila* IS26	Dif et al. (2022)
			*Enterobacter* sp. SA187	Andres-Barrao et al. (2017)
Cadaverine synthesis	Decarboxylation of l-lysine	*cadA*	*Stenotrophomonas* sp. 169	Ulrich et al. (2021)

**Polyamines** are present in nearly all organisms and are low molecular weight composites with aliphatic nitrogen structure (Kaushal and Wani, 2016). Polyamines like spermine, putrescine, and spermidine are functional agents of PGPB (Nascimento et al., 2020). Furthermore, spermidine production has been revealed to reduce the action of the ACCO gene in the ethylene synthesis pathway in tobacco (Nascimento et al., 2020).

**Ectoine** (1,4,5,6-tetrahydro-2-methyl-4-pyrimidinecarboxylic acid) accumulates in plants during salinity stress like other osmolytes and improves protein folding and protects biomolecules and even whole cells (Das et al., 2015; Sunita et al., 2020). Ectoine is synthesized by the products of *ectABC*. The *ectA*, *ectB,* and *ectC* genes code for diaminobutyric acid acetyltransferase, diaminobutyric acid aminotransferase, and ectoine synthase, respectively (Das et al., 2015; Sunita et al., 2020).

**Trehalose** is a non-reducing disaccharide, an αα -1,1-glucoside, comprising two molecules of α -glucose (Glick, 2012). Trehalose creates a gel phase that replaces water during cell dehydration (Forni et al., 2016). PGPB play a significant role in generating this osmoprotectant under salinity stress (Sunita et al., 2020). Five trehalose production pathways have been shown in bacteria consisting of treS, otsA/otsB (Tps/Tpp), treP, treT, and treY/treZ. Maltose is transformed to trehalose using trehalose synthase (*treS*) during the treS pathway. Trehalose is catalyzed in the otsA/otsB pathway by trehalose-6-phosphate synthase (*otsA*) and trehalose-6-phosphate phosphatase (*otsB*) (Nobre et al., 2008; Liu et al., 2016). In TreY/TreZ pathway, maltooligosyltrehalose synthase/hydrolase synthesizes trehalose. A trehalose phosphorylase (*treP*) catalyzes trehalose production in fungi and a few bacteria. A less common and lately revealed pathway applies a trehalose glycosyltransferring synthase (*treT*) (Nobre et al., 2008). *Rhizobium etli* involves the treYZ pathway to produce trehalose during osmotic stress (Mishra et al., 2018). UDP-glucose-4-epimerase or GALE, which is the product of gene *galE,* transforms reversibly UDP galactose to UDP glucose. UDP glucose participates in trehalose biosynthesis (Goyal et al., 2019). Genes linked to osmolyte synthesis in salinity stress-alleviating bacteria are presented in Table 5. The genes related to trehalose synthesis pathways have been identified in the genomes of 11 out of 20 assessed halotolerant PGPB and the genes related to ostA/ostB and treY/treZ pathways were the most prevalent pathways (Table 5).

RNA-Seq analysis of *Chromohalobacter salexigens* ANJ207 revealed the expression of genes related to betaine and choline transport systems and synthesis of glycine betaine, coline, and proline increased (Srivastava et al., 2022).

### 4.6 Molecular features of ion homeostasis

Bacteria contribute to toxic ion homeostasis that ameliorates plant tolerance under salinity. These bacteria decrease the absorption of toxic ions by controlling the expression of plants ion transporter and the construction of rhizosheaths through the production of exopolysaccharides (EPS) (Vaishnav et al., 2016; Ilangumaran and Smith, 2017). EPS binds cations, including Na^+^, and limits the entry of Na^*+*^ into roots (Etesami, 2020). PGPB contribute to the nutrient status in plants through microbial activities like organic acid excretion, phosphate solubilization, and siderophore synthesis, as mentioned in section 3-1. These nutrients reduce toxic ion accumulation (Vaishnav et al., 2016; Ilangumaran and Smith, 2017). PGPB enhance the K^+^ absorption by upregulating the K^+^ transporter, reducing the accumulation of Na^+^ in leaves and enhancing Na^+^ exclusion at the roots leading to an increased K^+^/Na^+^ ratio (Bhat et al., 2020; Shilev, 2020). Limiting the Na^+^ uptake at the root surface induces the HKT1 expression in shoots which assists the recirculation of Na^+^ from shoot to roots and aids in keeping an elevated K^+^/Na^+^ ratio in plants. The inoculation of *Helianthus annus* by *Bacillus subtilis* leads to the downregulation of HKT1/K^+^ transporter expression (Bhat et al., 2020).

### 4.7 Molecular basis of volatile organic compounds (VOCs)

Bacterial VOCs are species-specific metabolites sensed by other bacteria, insects, plants, animals, and microorganisms participating in cell-to-cell signaling and growth promotion (Kaushal and Wani, 2016, Ryu, 2015). Furthermore, their role in regulating bacterial motility, controlling virulence factors, and production of osmolytes, phytohormones, and siderophores are reported (Vaishnav et al., 2016). VOCs synthesis also affects the modulation of the HKT1/K^+^ transporter and controls the Na^+^ homeostasis pathway in plants (Vaishnav et al., 2016; Sunita et al., 2020). VOCs work as signals for a systemic reaction inside the same or neighboring plants (Bhat et al., 2020).

Acetoin and 2,3-butanediol are synthesized when two pyruvate molecules are compressed into acetolactate and transformed to acetoin *via* acetolactate decarboxylase, and lastly, acetoin reductase catalyzes 2,3-butanediol from acetoin (Liu et al., 2016; Suarez et al., 2019). *Hartmannibacter diazotrophicus* E19T genome contains the encoding gene of acetolactate synthase (*ilv*) but not genes related to acetolactate decarboxylase and acetoin reductase. Acetoin can be synthesized in E19T through a spontaneous decarboxylation of acetolactate into diacetyl, when oxygen is present, and then reduction of diacetyl to acetoin through the diacetyl reductase (product of *budC* gene) (Table 6) (Suarez et al., 2019).

**TABLE 6:** Genes related to volatile organic compounds (VOCs) synthesis in the genomes of 20 studied halotolerant PGPB.

Volatile organic compounds (VOC) synthesis	Product/Function	Genes	Bacteria	References
Acetoin synthesis	Acetolactate synthase, diacetyl reductase	*ilv, budC*	*Hartmannibacter diazotrophicus* E19T	Suarez et al. (2019)
	Acetolactate synthase, diacetyl reductase, Alpha-acetolactate decarboxylase	*ilv, budC, budA*	*Cronobacter muytjensii* JZ38	Eida et al. (2020)
	Alpha-acetolactate decarboxylase, Acetolactate synthase	*aldC, alsS*	*Bacillus megaterium* STB1	Nascimento et al. (2020)
	Acetolactate synthase, Alpha-acetolactate decarboxylase	*ilvHB, alsD*	*Bacillus flexus* KLBMP 4941	Wang et al. (2017)
	acetolactate synthase, zinc-containing alcohol dehydrogenase	*ilv, adh*	*Pseudomonas* sp. UW4	Duan et al. (2013)
Acetoin and 2,3-butanediol synthesis	Acetolactate synthase, acetoin reductase, acetolactate decarboxylase	*ilv, budC, butA, budA*	*Klebsiella* sp. D5A	Liu et al. (2016)
	acetolactate decarboxylase, Acetolactate synthase, Serine/threonine dehydratase, Ketol-acid reductoisomerase, Dihydroxy-acid dehydratase, aminotransferase, Acetolactate synthase	*alsD, ilv*	*Enterobacter roggenkampii* ED5	Guo et al. (2020)
	Acetolactate synthase, Alpha-acetolactate decarboxylase, diacetyl reductase	*ilvHB, alsD, and budC*	*Bacillus paralicheniformis* ES-1	Iqbal et al. (2022)

### 4.8 Molecular features of antioxidant defense mechanism

**FIGURE 5:**
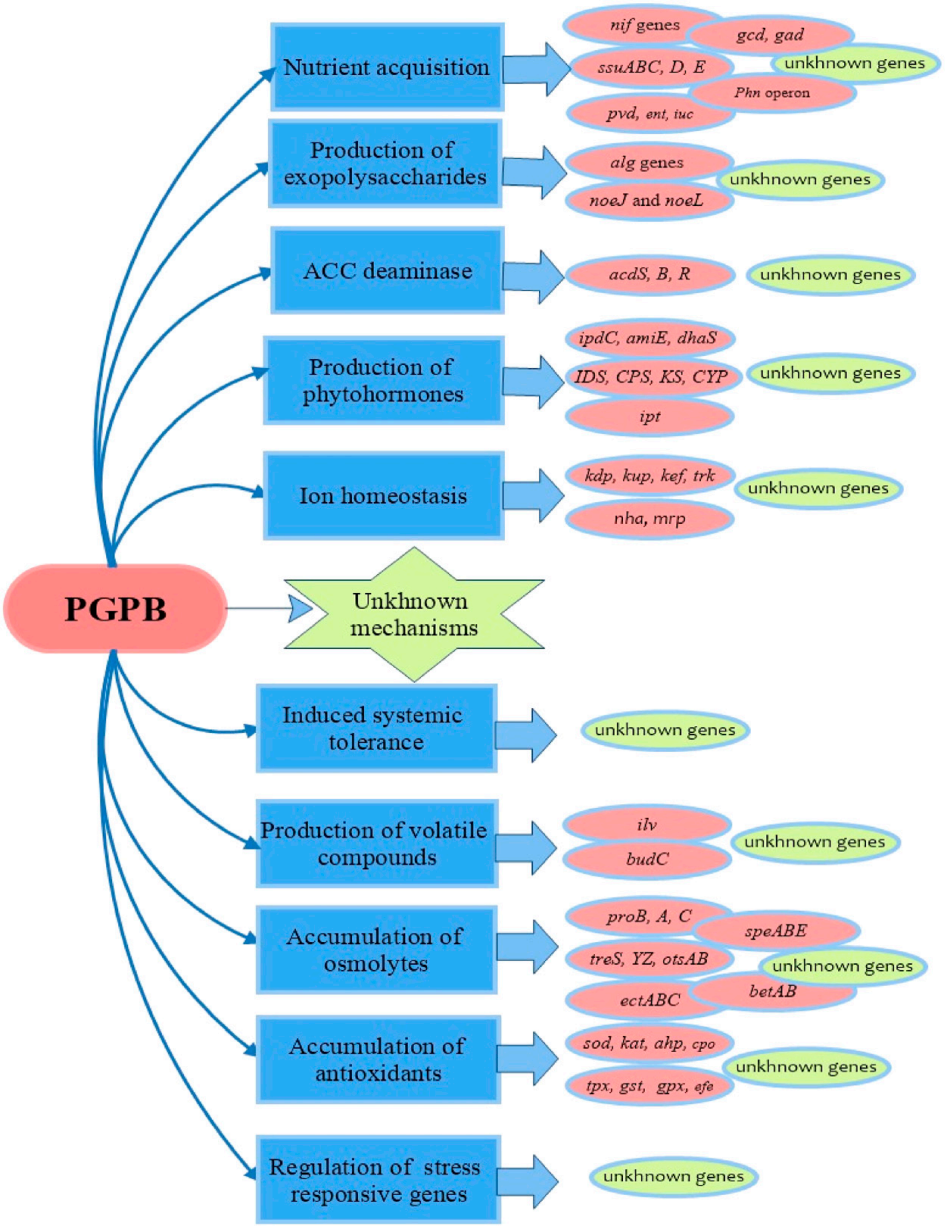
Mechanisms of salinity stress alleviation in plants by PGPB and so far assigned genes. There are some gaps and unknown mechanisms and genes that must be illustrated.

Plants are equipped with antioxidant defense systems, including enzymatic and non-enzymatic mechanisms against the harmful effects of ROS. Enzymatic elements comprise catalase (CAT), monodehydroascorbate reductase (MDHAR), superoxide dismutase (SOD), ascorbate peroxidase (APX), glutathione reductase (GR), and non-enzymatic compounds consist of ascorbate, cysteine, carotenoids, tocopherols, flavonoids, and glutathione (Arora et al., 2020; Kumar et al., 2020) (Table 7). PGPB can increase the functionality of antioxidant defense systems (Antar et al., 2021). *Bacillus thuringiensis* AZP2 induces SOD, CAT, and GR and enhances the tolerance of wheat to drought (Tiepo et al., 2020). Inoculated rice with *Trichoderma asperellum* and *Pseudomonas fluorescens* shows an increase in the action of APX, POD, CAT, and SOD (Bhat et al., 2020). Inoculation of *Arabidopsis* with *Enterobacter* sp. EJ01 caused higher APX activity (Mishra et al., 2018). *Trichoderma*, *Pseudomonas,* and their combination inoculation directly resulted in the upregulation of genes related to defense systems in plants, including phenylpropanoid (PAL), SODs, CAT, and APX (Singh et al., 2020). However, the mechanisms by which PGPB influence antioxidant enzymes are poorly understood (Figure 5).

**TABLE 7:** Identified genes related to antioxidant defense in the genomes of studied halotolerant PGPB.

Product/Functions	Genes	Bacteria	References
Alkylhydroperoxidase, chloroperoxidase, superoxidase dismutase, Glutathione S-transferase	*ahpCD, cpo, sodB, gstB*	*Hartmannibacter diazotrophicus* E19T	Suarez et al. (2019)
Superoxide dismutases, catalases, alkyl hydroperoxide reductases and thiol peroxidases, glutathione S-transferases, glutathione peroxidases, glutathione ABC transporter, gammaglutamate-cysteine ligase, glutathione synthetase, glutathione reductase and hydrolase, glutaredoxins and peroxiredoxins	*sodB, katE, katG, ahpCF, tpx, gst, gpx, gsiABCD, gshA, gshB, gor, ggt, grxABCD, BCP, ahpCF*	*Cronobacter muytjensii* JZ38	Eida et al. (2020)
Catalase, catalaseperoxidase, Superoxide dismutases, alkyl hydroperoxide reductase, peroxiredoxin, glutathione peroxidase, non-heme chloroperoxidase, glutathione S-transferase, superoxide oxidase	*KatE, katG, sod, ahpC, bcp, gpx, cpo, gst, cybB*	*Pseudomonas thivervalensis* SC5	Nascimento et al. (2021)
Peroxidase, catalase, superoxide dismutase, glutathione S-transferase, hydroperoxide	*efeB, katG, tpx, katE, catB, srp, sod, yncG, gst, yghU, ahpCF*	*Klebsiella* sp. D5A	Liu et al. (2016)
Superoxide dismutase, Catalase, Thiol peroxidase, chloroperoxidase, Glutathione peroxidase	*Sod, katE, tpx, cpo, gpx*	*Bacillus megaterium* STB1	Nascimento et al. (2020)
Glutathione synthase, glutathione-disulfide reductase, alkyl hydroperoxide reductase, alkyl hydroperoxide reductase, catalase, peroxidase, glucosylglycerol-phosphate synthase catalase, peroxidase	*gshB, gorA, ahpC ahpF, ggpS, katG*	*Stenotrophomonas* sp. 169	Ulrich et al. (2021)
Superoxide dismutase, Superoxide dismutase family protein, Catalase, Catalase-related peroxidase, Alkyl hydroperoxide reductase C, Peroxiredoxin, bromoperoxidase, chloroperoxidase, Pyridoxine/pyridoxamine 5′-phosphate oxidase, Glutathione peroxidase, Thioredoxin/glutathione peroxidase	*sodB, YojM, katE, srpA, AhpC, Bcp and OsmC, BPO-A2, Cpo, pdxH, garB BtuE*	*Stenotrophomonas rhizophila* IS26	Dif et al. (2022)
Glutathione peroxidase, Peroxiredoxin	*gpx, osmC*	*Enterobacter roggenkampii* ED5	Guo et al. (2020)
superoxide dismutase, catalase, peroxiredoxins, glutathione S-transferases, glutathione peroxidases	*Sod, katEN, ahpC, osmC, gts, btuE*	*Enterobacter* sp. SA187	Andres-Barrao et al. (2017)
superoxide dismutase, NAD(*P*)H oxidoreductase, flavohemoprotein, superoxide dismutase, catalase	*sodA1, ywrO, hmp, yojM, katE*	*Bacillus* sp. BH32	Belaouni et al. (2022)

The names of genera and species of bacteria and also the genes were presented in italic forms.

The comparisons of the expressed genes in *Burkholderia phytofirmans* PsJN from control plants and drought-stressed plants showed the genes related to glutaredoxin and alkyl hydroperoxide reductase were upregulated (Sheibani-Tezerji et al., 2015).

The transcriptome analysis of *Chromohalobacter salexigens* ANJ207 which is a halophilic PGPB showed the expression of genes related to catalase, thioredoxin reductase, oxidoreductase, methionine sulfoxide reductases, iron superoxide dismutase, peroxidase OsmC, peroxiredoxin, and an alkyl-hydroperoxide reductase, glutaredoxin, ferredoxin synthesis increased (Srivastava et al., 2022).

### 4.9 Molecular basis of stress-responsive genes

PGPB induce and up-regulate the expression of genes linked with tolerance to abiotic stresses in plants (Khan et al., 2020b; Sharma S. et al., 2021). Stress-responsive genes expression is mainly regulated *via* transcription factors and prompted through stress sensors that are exposed to complex control of phytohormones (Khan et al., 2020a). PGPB can augment tolerance of the plant to salt stress by regulating the expression of SOS genes, RAB18 (LEA), WRKY TFs, DRE (dehydration responsive element), RD29A, RD29B regulons of ABRE (ABA-responsive elements), besides transcription factor DRE binding proteins (DREB2b) (Chakdar et al., 2019; Etesami, 2020). PGPB also aids plants in reducing their cell water potential to continue absorbing water from saline soils by upregulating genes related to generating aquaporins. *Enterobacter* spp. upregulates salt stress-responsive genes including RAB18, DREB2b, RD29A, and RD29B in *Arabidopsis* in the salinity stress. TaMYB and TaWRKY genes expression are induced in inoculated wheat by *Dietzia natronolimnaea* (Kumar et al., 2020). *Bacillus amyloliquefaciens* SN13 upregulates SOS1, SERK1, EREBP, and NADP-Me2 genes and enhances the salt tolerance of rice (Chakdar et al., 2019).

### 4.10 Molecular features of exopolysaccharides (EPS) synthesis

Bacterial exopolysaccharides compose bound cell surface homo or hetero-polysaccharides in the form of capsule or slime, forming the skeleton of the biofilms. EPS production by PGPB under stress forms hydrophilic biofilms conferring desiccation protection, regulates nutrients and water flow across plant roots, binds to Na^+^ and decreases the bioavailability of the ion, aggregates root-adhering soils (RAS) and stabilizes soil aggregates (Mishra et al., 2018; Etesami, 2020; Jiang et al., 2021). The products of *noeJ* and *noeL* genes in *Azospirillum brasilense* Sp7 are mannose-6-phosphate isomerase, and GDP-mannose 4,6-dehydratase, which participate in EPS production. In *P. aeruginosa*, water scarcity condition induces *alg* genes of the alginate bioproduction gene cluster (Kaushal and Wani, 2016).

### 4.11 Molecular basis of nitric oxide signaling

Nitric oxide (NO) plays role in plant–bacteria interactions. NO has a dual effect (harmful and beneficial effects). Produced NO by PGPB, promotes plant growth and health and plays important roles in the response to environmental stress. NO influences root growth and developmental processes as a mediator in auxin-regulated signaling cascades, induces antioxidant system and scavenges directly ROS. On the other hand, NO is implicated in the pathogenesis of bacterial phytopathogens (Santana et al., 2017). NO dualistic nature depends on the NO level. Phytopathogens synthesize a high NO level and stimulate disease, while a lower level of NO synthesized by PGPR promote plant growth and increase plant tolerance (Santana et al., 2017).

Nitric oxide (NO) is an intermediate of denitrification process in bacteria. A membrane-bound nitrate reductase (Nar) or a periplasmic nitrate reductase (Nap) reduces nitrate to nitrite. Nitrite is reduced to NO either by a heme (NirS) or copper (NirK) containing nitrite reductase (Santana et al., 2017). NO is also synthesized in bacteria through nitric oxide synthases (NOS) which produce NO through the oxidation of l-arginine to l-citrulline. Moreover, ammonia-oxidizing bacteria produce NO by couples ammonia oxidation and denitrification. At first ammonium is oxidized to hydroxylamine by ammonium monooxygenase. Hydroxylamine oxidoreductase oxidizes hydroxylamine to nitrite. Finally, nitrite is reduced to NO by nitrite reductase (Molina-Favero et al., 2007; Santana et al., 2017).

Molina-Favero et al., 2008 showed the synthesized NO by *Azospirillum brasilense* Sp245 induced lateral and adventitious root development in inoculated tomato (Molina-Favero et al., 2008). Application of *Bacillus xiamenensis* ASN-1 and sodium nitroprusside (SNP) as a NO donor synergistically alleviated salinity stress in sugarcane by preserving the relative water content, gas exchange parameters, osmolytes, electrolyte leakage, and Na^+^/K^+^ ratio, modulating the antioxidant enzyme activities and stress-related gene expression (Sharma A. et al., 2021).

## 5 Discussion

Currently, due to the general requirement to feed a constantly growing world population, sustainable agriculture must be developed. This is despite the fact that the salinization of lands is one of the main obstacles to agricultural productivity. The application of salinity-tolerant PGPB and their metabolites improves the productivity of soils that are under salinity stress and has been a significant element in achieving the aim of food security and sustainability. Revealing the molecular mechanisms of action in PGPB and their interactions with plants is a crucial step toward targeting the use of PGPB as stress-alleviating biofertilizer. Accordingly, the number of genomes of rhizobacteria that were assembled, bioprojects, and articles on this subject in PubMed have increased 20, 7, and 6 times in 2020 compared to 2010, respectively. However, despite the development of new biotechnology tools and techniques, computational tools, and increasing studies that have been done on this issue, there are unknown gaps in molecular mechanisms of PGPB action because of the genetic and metabolic diversities of rhizobacteria and the intricacy of their interactions. Consequently, for evolving use of PGPB in sustainable agriculture, more studies must investigate the molecular aspect of PGPB action. To study genes, the PGPB genome analyzers need a better understanding of the genes and pathways related to plant growth promotion and stress alleviation that are known so far. In this study, the identified genes related to salinity stress mitigation and plant growth promotion in the genomes of 20 studied halotolerant PGPB are assessed. The genes related to the synthesis of IAA, siderophores, osmoprotectants, chaperons, ACC deaminase, and antioxidants, phosphate solubilization, and ion homeostasis are the most prevalent identified genes in the genomes of assayed halotolerant PGPB. The genes of *dnaJK*, *groEL*, *groES,* IPyA, and IAM pathways, and *entABCDEF* are the most commonly identified genes related to heat-shock proteins (HSPs), IAA, and enterobactin siderophore synthesis in the genomes of assessed halotolerant PGPB, respectively. Genes associated with the production of trehalose and glycine-betaine are the most prevalent identified osmoprotectants genes. In many of the assessed genomes of the halotolerant PGPB, there are two pathways of trehalose synthesis, and otsA/otsB, treY/treZ, and treS are the most common pathways. The presence of several trehalose biosynthesis pathways in the genomes of halotolerant PGPB can be due to the severe requirement to accumulate trehalose under stressful environmental conditions. There are several genes related to different antioxidant enzymes and non-enzymatic antioxidant mechanisms in the genomes of assessed halotolerant PGPB, which enabled them to tolerate stress. The identified genes related to gluconic acid synthesis (*gcd* and *pqq*) as the most prevalent agent for releasing phosphate and different phosphatases exist in the genomes of 11 of the assessed halotolerant PGPB. The existence of genes associated with phosphate solubilization in these halotolerant PGPB must be correlated to the decrease of the bioavailability of phosphorus in saline soil (Xie et al., 2022). Moreover, the genomes of assessed halotolerant PGPB contain the genes related to ion homeostasis, including *kdp* and *nha,* with high prevalence.

## 6 Conclusion

For the application of PGPB as stress-alleviating biofertilizers, determining the molecular aspects of action in PGPB and plant-bacteria interactions is a pivotal prerequisite. In this study, the identified genes and pathways related to plant growth promotion and stress alleviation in the genomes of 20 halotolerant PGPB (*Pseudomonas* and *Bacillus* genera, each with 20% prevalence as the most common genera) were assessed. The gene sets, pathways, and analog genes which have been identified in the genomes of assessed halotolerant PGPB can help the genome analyzers to mine the genomes of other plant-associated bacteria with higher accuracy. In addition, this data is useful for other omics and meata-omics studies. The genes related to synthesis of IAA (IPyA and IAM pathways), siderophores (based on the family of bacteria studied like *ent* and *pvd*), osmoprotectants (*otsAB, treS, treY*, *treZ,* and *betAB*), chaperons (*dnaJK, groES,* and *groEL*), ACC deaminase (*acds* and its homologs), and antioxidants (*sod, kat,* and *ahp*), phosphate solubilization (*gcd* and *pqq*), and ion homeostasis (*kdp* and *nha*) are the most prevalent attibuted genes in the genomes of these 20 halotolerant PGPB. Mentioned genes can be used as candidates for designing molecular markers to screen novel efficient PGPB for mitigating salinity stress. Genomics and metagenomics studies are only predictive of functional potential of bacteria. To determine the efficiency of PGPB, the expression of genes in them should be assayed in interaction with plants which reflect the inferiority of genomic interpretations *versus* interactomics with more realistic results. Moreover, due to the high intricacy of plant-bacteria interactions, and the genetic and metabolic diversities of plant-associated bacteria, further omics studies are needed to complete the gaps in molecular aspects of PGPB protective activities.

## 7 Future prospects

Further studies for illustrating the molecular detail of PGPB mechanisms of action will lead to the discovery of novel and diverse genes, pathways, and metabolites, and completion of gaps in the pathways. Furthermore, molecular analysis assists in identification of silent genes under lab conditions (because of lack of natural triggers or signals), introducing safety assessment markers (with analysis of presence or absence of pathogenic genes), easy and fast screening of more PGPB strains with the utilization of gene markers, finding strains with multiple capabilities such as bioremediation, neutralizing biotic/abiotic stress, and optimization of applying PGPB as biofertilizer. Furthermore, considering that about 1% of soil bacteria are culturable, metagenomic analysis of the rhizospheric bacteria will provide access to new and rich gene repositories for plant protection.) In combination with findings from applying other meta (omics) approaches in interaction studies between different bacteria, plants, and natural environment will unreveal the unknowns of pathways and mechanisms of actions. Molecular aspects of how PGPB cause induced systemic resistance (ISR) and regulate stress responsive genes are among the mechanisms that need to be studied more.

Accordingly, future advances in sequencing, mass spectrometry, and other metabolomics technologies, annotating and predicting platforms, and databases will facilitate the identification of molecular aspects with high throughput and coverage depth to open the current bottleneck in soil interactomics and metaphenomics.

## Abbreviations

PGPB, plant growth-promoting bacteria; IAA, indole-3-acetic acid; NGS, next-generation sequencing; ECF, extracytoplasmic function; HSPs, Heat-shock proteins; KSB, K-solubilizing bacteria; NRPSs, non-ribosomal peptide synthetases; PSB, Phosphate-solubilizing bacteria; GA, gluconic acid; PQQ, pyrrolo-quinolone quinine; NSAPs, non-specific acid phosphatases; ABA, Abscisic acid; IPyA, indole-3-pyruvate; IAM, indole-3-acetamide; GAs, Gibberellins; SA, Salicylic acid; ACC, 1-aminocyclopropane-1-carboxylic acid; ACCO, ACC oxidase; ACCS, ACC synthase; ACCD, ACC deaminase enzyme; VOCs, volatile organic compounds; GB, Glycine betaine; EPS, exopolysaccharides; CAT, catalase; MDHAR, monodehydroascorbate reductase; SOD, superoxide dismutase; APX, ascorbate peroxidase; GR, glutathione reductase; RAS, root-adhering soils.
